# Evaluation of Seattle’s sweetened beverage tax on tax support and perceived economic and health impacts

**DOI:** 10.1016/j.pmedr.2022.101809

**Published:** 2022-04-30

**Authors:** Vanessa M. Oddo, Melissa A. Knox, Lina Pinero Walkinshaw, Brian E. Saelens, Nadine Chan, Jessica C. Jones-Smith

**Affiliations:** a University of Illinois Chicago, College of Applied Health Sciences, Department of Kinesiology and Nutrition, Chicago, IL, USA; b University of Washington, Department of Economics, Seattle, WA, USA; c University of Washington School of Public Health, Department of Health Systems and Population Health, Seattle, WA, USA; d Seattle Children’s Research Institute, Seattle, WA, USA; e University of Washington School of Medicine, Department of Pediatrics, Seattle, WA, USA; f Public Health - Seattle and King County, Seattle, WA, USA; g University of Washington School of Public Health, Department of Epidemiology, Seattle, WA, USA

**Keywords:** Sugar sweetened beverages, SSB taxes, Health policy, Nutrition policy, Norms and attitudes

## Abstract

It is important to understand whether the publics’ attitudes towards sugary beverage taxes (SBT) change after tax implementation to ensure the long-term success of tax policies. Seattle’s SBT went into effect on January 1, 2018. We administered a mixed-mode survey to adults in Seattle and comparison areas, pre- and 2-years post-tax, to evaluate the impact of the SBT on 1) tax support and 2) perceived tax impacts (N = 2,933). Using a difference-in-differences approach, we employed adjusted income-stratified modified Poisson models to test the impacts of the tax on net changes in attitudes in Seattle versus the comparison areas, pre- to post-tax. Among lower-income individuals in Seattle, support for the tax increased by 14% (PR_DD_: 1.14; 95% CI: 1.08, 1.21) and there was a 20% net-increase in the perception that the SBT would positively affect the economy (PR_DD_: 1.20; 95% CI: 1.05, 1.39), compared to changes in the comparison areas. Among higher-income individuals in Seattle, support for the tax was not different (PR_DD_: 0.93; 95% CI: 0.70, 1.22) pre- to post-tax, but there was a net-increase in the perception that the tax would have negative effects on small businesses (PR_DD_: 1.44; 95% CI: 1.03, 2.00) and family finances (PR_DD_: 1.86; 95% CI: 1.09, 3.19). After living with the tax for 2-years, support for the tax increased among lower-income individuals in Seattle. Tax support was high and unchanged among higher-income individuals, but overall attitudes became more negative. Policy makers should consider investing in ongoing campaigns that explain the benefits of SSB taxes and revenues.

## 1 Introduction

Sugar-sweetened beverage (SSB) consumption is an important contributor to adverse health outcomes such as obesity, type 2 diabetes and cardiovascular disease (Malik et al., 2013, Malik et al., 2010, Woodward-Lopez et al., 2011). As such, 8 U.S. cities have sought to reduce SSB consumption by levying taxes on sweetened beverage distribution. Most prior U.S. studies report that sweetened beverage taxes (SBT) increase the price of SSBs and result in reductions in the volume sold of taxed beverages (Falbe et al., 2016, Jones-Smith et al., 2020, Powell et al., 2020, Powell and Leider, 2020, Roberto et al., 2019, Silver et al., 2017), which can reduce consumption (Falbe et al., 2016, Zhong et al., 2018). However, prior studies have yet to investigate the extent to which the publics’ attitudes towards SBTs change after a tax is implemented; understanding whether attitudes change is important for the long-term success and future implementation of SBTs.

In previous work, we found that a majority of Seattle residents supported the SBT, prior to implementation (Oddo et al., 2019). However, there are several mechanisms through which attitudes could change after individuals lived with the tax, including “fatigue” related to having to pay the tax, the potential inconvenience of cross-border shopping to avoid SSB price increases, and/or potentially unfounded concerns that a local business closure was due to the tax (Marinello et al., 2021, Marinello et al., 2021). It is also likely that awareness of the SBT increased after implementation as many Seattle stores noted the tax on price tags (e.g., price tags stated “plus beverage tax” or “includes sweetened beverage tax”). Additionally, both pro- and anti-tax messaging and media about the consequences of SBTs tend to be prominent post-implementation and this could shift tax support (Barry et al., 2013, Chriqui et al., 2020, Curry et al., 2018, Donaldson et al., 2015, Gollust et al., 2017). For example, in Illinois, anti-tax media and public pushback because of tax fatigue led to repeal of their SBT after implementation (Chriqui et al., 2020). In Seattle, there was a grassroots-led effort to conduct pro-tax outreach in lower-income communities and communities of color. At the same time, after tax implementation, Seattle news organizations mostly focused on the potential negative impacts of the tax on small businesses and there was a large anti-beverage tax campaign in Seattle that accompanied a Washington state bill that preempted the ability of local jurisdictions to tax SSBs (Jones-Smith et al., 2021, O’Sullivan, 2018, Zenone and Kenworthy, 2021).

Additionally, in prior work we found that lower-income versus higher-income Seattle residents were more concerned about the potential financial consequences of the tax (Oddo et al., 2019). Prior literature provides preliminary descriptive evidence of an association between individual sociodemographic characteristics and support for and attitudes towards SBTs (Donaldson et al., 2015, Curry et al., 2018, Rivard et al., 2012, Julia et al., 2015, Gollust et al., 2014, Altman et al., 2021). In particular, a recent study of SBTs in the Bay Area found that those with lower levels of education and non-Hispanic Black populations were less likely to believe that SBTs benefited the community and children’s health; authors attributed findings, in part, to limited ongoing communication regarding the tax and revenue use (Altman et al., 2021). However, this study only surveyed individuals post-tax, so it is unclear whether attitudes changed in response to the tax itself.

We improve upon this descriptive evidence by employing a quasi-experimental design to investigate whether individuals’ attitudes changed after living with the tax and tax-messaging in Seattle for 2-years. Given that tax support and perceptions may differ by sociodemographics and the ongoing debate that SBTs are regressive, we investigated this association using income-stratified models.

## 2 Methods

### 2.1 Overview

Seattle’s SBT went into effect on January 1, 2018 and large distributors began to pay a 1.75 cents per ounce excise tax. We used a difference-in-differences estimation approach to test the change in attitudes towards the tax in Seattle versus a comparison area, pre- to 2-years post-tax implementation. The comparison area was comprised of individuals from Minneapolis, MN, Rockville, MD, Bethesda, MD and Arlington, VA. We initially evaluated 600 census places and narrowed to those that were well-matched on racial/ethnic composition and education, and not actively considering a tax. We then selected cities that were similar to Seattle on the following characteristics: total population, population density, percent of the population that was non-Hispanic white, non-Hispanic Black, non-Hispanic Asian, non-Hispanic Other, Hispanic, and with a college degree or higher, household size, per capita income, median household income, and percent of population that voted for the Democratic presidential nominee in 2016.

### 2.2 Data collection and sample

We conducted a mixed-mode survey (telephone and online) that was fielded by trained interviewers at the survey research firm, Ironwood Insights Group, LLC. Baseline data were collected pre-tax implementation (October-December 2017) and endline data were collected 2-years post-tax implementation (September-November 2019). Phone and online versions of the survey were offered in English and Spanish and the online version was additionally offered in Vietnamese. All Seattle and comparison area residents aged 18 + were eligible for inclusion. Additional inclusion criteria included answering the screener questions on household income and race/ethnicity.

We aimed to recruit cross-sectional samples that had a similar racial/ethnic distribution to the 2017 American Community Survey (ACS) sample in Seattle and comparison areas. Based on previous literature, our *a prori* hypothesis was that changes in attitudes could differ by income-level (Donaldson et al., 2015, Curry et al., 2018, Rivard et al., 2012, Julia et al., 2015, Gollust et al., 2014, Altman et al., 2021). Therefore, we also aimed to recruit a sample that was powered to test for differences by income status (lower-income: < 260% of the Federal Poverty Level [FPL] versus higher-income: ≥260% FPL). At baseline, we recruited 851 participants living in Seattle (456 higher-income, 395 lower-income) and 863 living in the comparison areas (453 higher-income, 410 lower-income). At the post-tax measurement, we recruited 800 total participants living in Seattle (442 higher-income, 358 lower-income) and 800 participants in the comparison areas (444 higher income, 356 lower-income).

### 2.3 Analytic sample

We recruited 3,314 individuals. However, we identified 93 individuals whose screener question related to FPL classification was inconsistent with their report of household income, asked later in the survey. We could not determine which question accurately captured these individuals’ income-level, so they were excluded. Additionally, 228 people were excluded due to missing information on variables used in creation of the propensity score weight (described below). This resulted in an initial analytic sample of 2,993 individuals. Additionally, as described below, in some models “don’t know” responses were coded as missing values and excluded from analyses.

### 2.4 Primary independent variable

The SBT was implemented on January 1, 2018 in Seattle. Comparison areas were not subject to a tax.

### 2.5 Primary dependent variables

Survey questions were developed and adapted based on prior research and polling (Gollust et al., 2017, Gollust et al., 2014, Niederdeppe et al., 2014). Detailed in files S1 and S2, we first described the tax (or SBTs generally) and explained how the tax revenue would be used. Then, Seattle residents were asked about their attitudes in relation to the tax implemented in January 2018 (e.g., the tax will improve public health in Seattle). Comparison area residents were asked about SBTs generally (e.g., these taxes would improve public health). Questions were piloted by the research firm prior to the survey launch.

We explored several dependent variables, broadly grouped into 2 categories: 1) perceived economic and health impacts of the tax and 2) tax support.

#### 2.5.1 Perceived economic and health impacts of the tax

First, we created an attitudes index score to summarize individuals’ overall perceptions related to the possible impacts of the tax(es), comprised of seven questions on small businesses, the economy broadly, job loss, family finances, the well-being of low-income people and people of color, public health and child well-being. Participants were read two statements and asked which statement was “much” or “somewhat” closer to their perception. For example, participants were asked whether the first statement *“This tax will result in job loss”* was much or somewhat closer to their own view, compared to the second statement “*This tax will not result in job loss”.* “Much” or *“*somewhat” closer responses for each statement were collapsed. Participants were also given the option to report “don’t know”. We assigned a 1 if tax impacts were reported as positive/beneficial, a 0 if they responded that they “don’t know,” and a −1 if the tax impacts were reported as negative/detrimental (range: −7 to 7). A lower score was interpreted to mean that the tax impacts were negative/detrimental.

Second, we investigated the question-specific economic and health impacts of the tax in 7 separate regression models. In these models, “don’t know” responses were coded as missing values and excluded from analyses.

#### 2.5.2 Tax support

Third, we queried whether participants approved or disapproved of the tax(es) itself, using a four-category Likert scale: strongly approve, somewhat approve, somewhat disapprove, and strongly disapprove. We collapsed the responses into “approve” and “disapprove” and “don’t know” responses were excluded from analyses.

### 2.6 Population and propensity score weights

First, we created population weights using the raking method (Deville et al., 1993) to weight results to the Seattle and comparison areas population totals, based on the 5-year ACS (2013–2017) for race/ethnicity, gender, age, educational attainment, and household income. Second, we created propensity score weights, because differences in the composition of the four groups (i.e., baseline Seattle, baseline comparison, endline Seattle, endline comparison) and changes in their composition over time might create the appearance of a trend in the outcomes, where one does not exist (Stuart et al., 2014). We created income-stratified propensity score weights that weighted the baseline comparison, endline Seattle, and endline comparison samples to match the baseline Seattle sample on several observed covariates (race/ethnicity, educational attainment, age, gender, marital status, political affiliation, survey mode) (Ridgeway et al., 2015). We then multiplied the population weight by the propensity score weight and trimmed the weights to the 99th percentile.

We estimated the standardized absolute mean difference, by income, across all the covariates to check the balance of sample characteristics after weighting. We observed a lower average standardized difference and variance ratios closer to 1 when employing the combination population-propensity score weights (versus unweighted); once weighted, the groups were similar based on measured covariates (Tables S1a and b) (Austin, 2009).

### 2.7 Statistical analyses

We employed survey weighted income-stratified difference-in-differences linear and modified Poisson regression models, with robust standard errors (Zou, 2004), to estimate the extent to which perceived tax impacts and tax support in Seattle changed above and beyond the change in the comparison areas, over 2-years. Exponentiated coefficients from the modified Poisson models were interpreted as prevalence ratios. All regression models were weighted using the combination population-propensity score weight and controlled for race/ethnicity, education, household income, age, gender, marital status, political affiliation, and survey mode, resulting in doubly robust estimates.

Robust standard errors were clustered at the city-level. Statistical analyses were performed using Stata 15.1 (StataCorp LP, College Station). Alpha was set to 0.05. The University of Washington Institutional Review Board determined that this study was exempt from review.

## 3 Results

### 3.1 Descriptive statistics

Approximately half of the weighted sample, in both Seattle and comparison areas, was female and identified as a Democrat (Table 1). The weighted sample was ∼ 65% non-Hispanic white, 7% non-Hispanic Black/African American, 14% non-Hispanic Asian, 7% non-Hispanic of another race, and 7% Hispanic. Approximately 60% of the weighted sample were higher income (≥260% FPL) and had a college degree or higher. About 20% of the weighted sample were aged 18–30 years, 60% were aged 30 to 65, and 15% were aged ≥ 65 years.

**Table 1:** Selected demographic characteristics of Seattle and the comparison area samples.^a^

	Seattle				Comparison			
	Pre-Tax (N = 781)		Post-Tax (N = 738)		Pre-Tax (N = 729)		Post-Tax (N = 745)	
	N	%	N	%	N	%	N	%
Gender								
Male	321	50.7%	313	49.9%	399	48.7%	241	50.4%
Female	460	49.3%	425	50.1%	330	51.3%	504	49.6%
Race/Ethnicity								
Non-Hispanic White	550	64.8%	509	64.3%	464	64.6%	472	66.3%
Non-Hispanic Black	52	7.0%	52	8.5%	61	7.2%	96	6.0%
Non-Hispanic Asian	62	14.3%	70	13.9%	73	13.9%	69	13.5%
Non-Hispanic Other^b^	70	7.0%	55	6.8%	29	6.4%	54	8.4%
Hispanic	47	7.0%	52	6.5%	102	7.9%	54	5.8%
Age of Respondent								
18–30 years old	129	20.1%	135	20.8%	146	19.7%	187	21.5%
31–40 years old	145	22.5%	150	23.2%	168	24.3%	158	22.7%
41–50 years old	123	19.8%	119	18.8%	101	18.4%	90	15.9%
51–64 years old	155	23.4%	183	22.1%	141	24.4%	136	23.5%
65 + years old	229	14.2%	151	15.1%	173	13.1%	174	16.3%
Highest Level of Education								
Some College	281	36.3%	312	36.4%	313	39.8%	314	37.8%
Completed College	500	63.7%	426	63.6%	416	60.2%	431	62.2%
Income Relative to FPL								
Low Income: < 260%	355	38.3%	317	35.8%	313	43.1%	327	34.5%
High Income: ≥ 260%	426	61.7%	421	64.2%	416	56.9%	418	65.5%
Political Affiliation								
Democrat	440	53.6%	401	53.7%	317	54.8%	382	52.1%
Independent	225	29.4%	208	28.8%	225	27.4%	182	29.2%
Republican	63	8.9%	71	9.7%	119	8.8%	92	9.7%
Other	11	1.8%	25	1.6%	14	2.1%	13	2.4%
Don’t know	42	6.3%	33	6.1%	54	6.9%	76	6.7%
Survey Mode								
Web	419	57.9%	503	58.8%	557	59.2%	611	61.1%
Phone	362	42.1%	235	41.2%	172	40.8%	134	38.9%

a N is unweighted to show the sample size whereas percentages (%) are weighted using the population weight X propensity score weight.

b Native Hawaiian or Other Pacific Islanders, American Indian and Alaska Natives, and those reporting two or more races are categorized as non-Hispanic Other.

#### 3.1.1 Lower-income

Table 2 details the descriptive responses to the questions about tax impacts and support. The proportion of lower-income individuals in Seattle that perceived that the SBT would result in job loss was 27.3% pre-tax versus 30.9% post-tax, contrary to the trends in the comparison areas (36.3% pre-tax versus 32.2% post-tax). Among lower-income individuals in Seattle, 44.1% and 54.8% perceived that the SBT would have negative effects on small businesses pre- and post-tax, respectively. In the comparison area, 50.3% perceived there would be negative effects of SBTs broadly on small businesses pre-tax, compared to 54.5% post-tax. In Seattle, 52.1% perceived that the tax would improve public health pre-tax versus 57.5% post-tax; trends were similar in the comparison areas at both timepoints. Among lower-income individuals, a slim majority of supported the SBT pre- (52.6%) and post-tax (50.5%) in Seattle. In the comparison areas, tax support was 55.3% pre-tax and 47.3% post-tax. Additional descriptive data with the “don’t know” responses are detailed in Tables S2.

**Table 2:** Descriptive Pre-tax to Post-tax Prevalences in Perceptions of Sweetened Beverage Taxes in Seattle, Washington, and the Comparison areas, by Income.

	Lower-Income^a^				Higher-Income^a^			
	Seattle		Comparison		Seattle		Comparison	
	Pre-tax^b^	Post-tax^b^	Pre-tax^b^	Post-tax^b^	Pre-tax^b^	Post-tax^b^	Pre-tax^b^	Post-tax^b^
Support for sugary beverage tax(es)	52.6%	50.5%	55.3%	47.3%	64.8%	62.5%	60.0%	61.2%
Tax(es) will/would have negative effects on small businesses	44.1%	54.8%	50.3%	54.5%	41.1%	55.4%	46.5%	45.3%
Tax(es) will/would have a positive effect on the economy	53.3%	61.7%	54.3%	53.0%	59.1%	61.5%	57.3%	51.8%
Tax(es) will/would result in job loss	27.3%	30.9%	36.3%	32.2%	23.7%	31.4%	29.6%	23.0%
Tax(es) will/would have a negative impact on family's finances	27.8%	31.9%	32.5%	33.1%	13.7%	24.6%	20.6%	18.9%
Tax(es) will/would have a positive impact on people with low-income and people of color’s health/well-being	50.8%	46.9%	53.4%	50.1%	56.0%	50.0%	43.7%	48.2%
Tax(es) will/would improve public health	52.1%	57.5%	55.4%	55.0%	62.8%	61.9%	60.0%	62.2%
Tax(es) will/would improve child wellbeing	57.5%	60.1%	58.6%	58.7%	63.9%	65.4%	62.5%	66.0%

a Lower income is defined as < 260% FPL. Higher income is defined as ≥ 260% FPL.

b Percentages (%) are weighted using the population weight X propensity score weight.

#### 3.1.2 Higher-income

In Seattle, 41.1% of individuals perceived that the SBT would have negative effects for small businesses pre-tax versus 55.4% post-tax; trends were similar (∼45%) at both timepoints in the comparison areas. Among higher-income individuals in Seattle, 23.7% perceived that the SBT would result in job loss pre-tax, versus 31.4% post-tax. In the comparison area, 29.6% and 23.0% perceived that SBTs broadly would result in job loss pre- and post-tax, respectively. The perception that the tax(es) would negatively affect family finances was 13.7% and 24.6% in Seattle and 20.6% and 18.9% in the comparison areas, pre- versus post-tax, respectively. Among higher-income individuals, tax support was high, ∼60% in both Seattle and the comparison areas at both timepoints.

### 3.2 Difference-in-differences estimation

#### 3.2.1 Overall attitudes index score

Among lower-income individuals, the combined attitudes index score related to tax impacts did not change in Seattle versus the comparison areas, pre- to 2-years post-tax (β_difference-in-difference[DD]_: −0.03; 95% Confidence Interval [CI]: −0.34, 0.28) (Table 3). However, among higher-income individuals, net attitudes related to tax impacts in Seattle versus the comparison became more negative (β_DD_: −1.10; 95% CI: −1.82, −0.38).

**Table 3:** Adjusted Pre-tax to Post-tax Changes in Support for and Overall Perceptions of Sweetened Beverage Taxes in Seattle, Washington, relative to the Comparison areas, by Income.

	Lower-Income^a^		Higher-Income^a^	
	N	PR_DD_ or β_DD_ (95% Confidence Interval)	N	PR_DD_ or β_DD_ (95% Confidence Interval)
Support for the sweetened beverage tax(es)^b^, ^c^	1276	**1.14 (1.08, 1.21)**	1657	0.93 (0.70, 1.22)
Overall attitudes impacts score^c^, ^d^	1367	−0.03 (−0.34, 0.28)	1732	**−1.10 (**−**1.82, −0.38)**

DD = Difference-in-differences; PR = prevalence ratio.

a Lower income is defined as < 260% FPL. Higher income is defined as ≥ 260% FPL. Boldface indicates statistical significance (p < 0.05).

b Estimated using modified Poisson models with difference-in-differences estimation.

c Estimates are weighted to be representative of the populations in each area and are propensity score weighted to control for confounding by demographic differences across city and time point. Models also control for race/ethnicity, educational attainment, age, gender, marital status, political affiliation, and survey mode. Standard errors are clustered at the city-level.

d Estimated using linear models with difference-in-differences estimation. The tax impacts score is comprised of the following questions: child well-being, public health, small businesses, the economy, job loss, family finances, and impacts on people with lower-income and people of color. We assigned a 1 if the impact of the tax was perceived as positive/beneficial, a 0 if they responded that they “don’t know,” and a −1 if the tax was perceived as negative/detrimental (score range: −7 to 7). A lower score was interpreted to mean that perceptions about the tax impacts were negative.

### 3.3 Question-specific economic and health impacts

#### 3.3.1 Lower-income

**Fig. 1:**
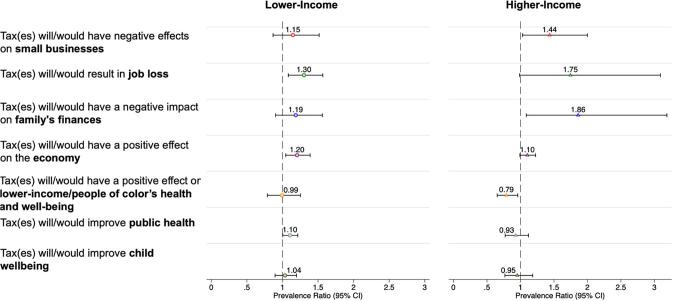
Adjusted Pre-tax to Post-tax Changes in Item-Specific Perceptions of Sweetened Beverage Taxes in Seattle, Washington, relative to the Comparison areas, by Income ^a,b^CI = confidence interval^a^Lower income is defined as < 260% FPL. Higher income is defined as ≥ 260% FPL. ^b^Estimates presented are the difference-in-differences coefficients, using modified Poisson models. They are weighted to be representative of the populations in each area and are propensity score weighted to control for confounding by demographic differences across city and time point. Models also control for race/ethnicity, educational attainment, age, gender, marital status, political affiliation, and survey mode. Standard errors are clustered at the city-level.

Among lower-income individuals in Seattle versus the comparison areas, there was a 20% net-increase in the perception that the SBT would have positive effect on the economy (PR_DD_: 1.20; 95% CI: 1.05, 1.39) and a 10% net-increase in the perception that the SBT would improve public health (PR_DD_: 1.10; 95% CI: 1.00, 1.22) (Fig. 1). However, there was also a 30% net-increase in the perception that the SBT would result in job loss (PR_DD_: 1.30; 95% CI: 1.08, 1.57). Among lower-income individuals, there were no statistically significant changes in perceptions towards tax impacts on small businesses, family finances, marginalized populations or on child well-being in Seattle versus the comparison areas, pre- to post-tax.

#### 3.3.2 Higher-income

Among higher-income individuals in Seattle versus the comparison areas, the perception that the tax would have negative effects on small businesses (PR_DD_: 1.44; 95% CI: 1.03, 2.00) and family finances (PR_DD_: 1.86; 95% CI: 1.09, 3.19) significantly increased pre- to post-tax. Correspondently, in Seattle versus comparison areas, there was a 21% net-decrease in the perception that the SBT would have a positive impact on marginalized populations’ health and well-being (PR_DD_: 0.79; 95% CI: 0.65, 0.96). Net-changes in attitudes towards the economy broadly, job loss, and effects on health and well-being were not statistically significant among higher-income individuals.

### 3.4 Tax support

Among lower-income individuals, support for the SBT increased by 14% (PR_DD_: 1.14; 95% CI: 1.08, 1.21) in Seattle compared to the change in support in the comparison areas, in the 2-years post-tax (Table 3). Among higher-income individuals, the net-change in support for the tax was not different pre- to post-tax (PR_DD_: 0.93; 95% CI: 0.70, 1.22).

## 4 Discussion

This is the first quasi-experimental study to investigate whether individuals’ attitudes changed as a result of living with the tax and tax-messaging in Seattle for 2-years. Among lower-income individuals, support for the tax increased by 14% in Seattle, relative to the change in the comparison area. Tax support was high at both timepoints (∼60%) among higher-income individuals. Among lower-income individuals in Seattle, there was a 20% net-increase in the perception that the SBT would have positive effect on the economy and a 10% net-increase in perception that the tax improves public health, pre- to 2-years post-tax. But, among lower-income individuals, there was also a net-increase in the perception that the tax would result in job loss. Despite maintaining higher and stable support for the tax, higher-income individuals’ overall attitudes related to possible tax impacts became more negative in Seattle versus the comparison areas, in the 2-years post-tax. We also observed a 44% and 86% net-increase in the perception that the tax would negatively impact small businesses and family finances, respectively, among this group. At the same time, many perceptions in Seattle versus the comparison area did not change.

The increase in tax support among lower-income individuals and overall perceptions becoming more negative among higher-income individuals may stem from the messaging environment in Seattle, which is generally supported by prior literature (Julia et al., 2015, Donaldson et al., 2015, Curry et al., 2018, Barry et al., 2013, Gollust et al., 2017, Chriqui et al., 2020, Bosire et al., 2020, Jou et al., 2014, Asada et al., 2022, Falbe et al., 2020). There was no city-government supported pro-tax or educational campaign in Seattle, after tax implementation. However, there were several other campaigns that might help explain the differences by income-level. Consistent with the increased support among lower-income individuals in Seattle, there were several grassroots organizations that conducted outreach in lower-income communities to provide information about the rationale for the SBT. Leveraging and strengthening existing community partnerships for campaigns to raise awareness and counter misinformation has been found to be an important component of SBT implementation (Asada et al., 2022). Additionally, by ordinance mandate, a Community Advisory Board advised the City of Seattle regarding revenue distribution, to ensure revenues were used to fund programs that would benefit lower-income populations (e.g., the Fresh Bucks program) (Moss et al., 2019). Higher-income individuals in Seattle perceiving tax impacts more negatively, after 2-years, could be explained by greater exposure to anti-tax messaging. In our prior work, we found that a higher proportion of people in Seattle (47%) versus the comparison area (29%) reported seeing more negative messaging about SBTs. Moreover, among Seattle residents, fewer higher-income respondents reported seeing positive messages or media related to the tax (22% versus 32% of lower-income individuals) (Jones-Smith et al., 2021). Seattle news organizations mostly focused on the potential negative impacts on small businesses and in the Summer of 2018, there was a large anti-beverage tax campaign in Seattle that accompanied a Washington State preemption bill; estimates suggest that the beverage industry spent approximately $20 million on the “Yes to Affordable Groceries” anti-tax campaign (Crosbie et al., 2021). These findings are generally consistent with studies focused on SBT implementation, which suggest messaging is important for the passage and long-term success of these taxes (Asada et al., 2022, Falbe et al., 2020). Findings from Berkeley suggested that early and robust outreach to the public about the tax and programs funded may promote public support and pro-tax campaigns may be important in mitigating well-funded repeal efforts or post-enactment lawsuits (Falbe et al., 2020). Likewise, in Oakland, a pro-tax coalition and campaign were important for counteracting industry attacks (Asada et al., 2022). In combination with evidence from Illinois, Berkeley, and Oakland, our findings suggest that if officials want to sustain broad public support for these taxes, they should invest in ongoing campaigns that explain the benefits of SBTs and provide information about how tax revenues are being used (Chriqui et al., 2020, Altman et al., 2021, Asada et al., 2022, Falbe et al., 2020).

Additionally, our findings are generally consistent with the few prior cross-sectional studies suggesting that support for SBTs and the perceived benefits and tax support may differ by demographic characteristics (Donaldson et al., 2015, Curry et al., 2018, Rivard et al., 2012, Julia et al., 2015, Gollust et al., 2014, Altman et al., 2021). However, we found that higher-income individuals’ attitudes became more negative when living with the SBT and related tax-messaging in Seattle; this is somewhat in contrast with prior studies finding that those with lower-education, which is correlated with lower-income, perceived fewer benefits of SBTs on health and the community (Julia et al., 2015, Altman et al., 2021). Contrary findings may be related to the methodological limitations, as these studies largely pre-date the implementation of SBTs and do not assess net-changes over time, using a quasi-experimental design.

This study had some limitations. First, this was a repeated cross-sectional survey, so we cannot rule out the possibility of unmeasured factors that could have occurred in either Seattle or comparison areas, which could introduce bias. However, we do employ a quasi-experimental design and propensity score weights to account for compositional differences in the repeat cross-sectional samples over time. Second, the proportion of the sample completing the survey online rather than the phone was larger in both Seattle and the comparison areas at the post-tax timepoint, which could result in less social desirability bias than in the baseline survey. However, we control for survey mode and include mode in the creation of our weight to better account for the possibly of modal bias. Third, generalization of our results to other cities may be limited. Fourth, we did not explicitly approach this research question through a policy implementation science lens; however, doing so could further contribute to our understanding of the circumstances under which SBTs are more likely to be implemented and help ensure full implementation of SBTs once adopted (Emmons et al., 2021). Finally, although survey questions were piloted, our survey items were not explicitly validated, and the results may be specific to the use of this measure.

## 5 Conclusions

After living with the tax for 2-years, support increased among lower-income individuals in Seattle, on average. Despite maintaining higher and stable support for the tax, higher-income individuals overall perceptions became more negative over time. This may in part be attributed to messaging. Policy makers should consider investing in ongoing pro-tax campaigns or in dissemination of positive tax outcomes when implementing an SBT in order to maintain public support for the policy and avoid repeal, which jeopardizes the intended public health and revenue generating goals.

## 6 Financial Disclosures

No financial disclosures were reported by the authors of this paper.

### CRediT authorship contribution statement

**Vanessa M. Oddo:** Conceptualization, Data curation, Formal analysis, Methodology, Writing – original draft. **Melissa Knox:** Data curation, Methodology, Writing – review & editing. **Lina Pinero Walkinshaw:** Project administration, Writing – review & editing. **Brian E. Saelens:** Conceptualization, Methodology, Writing – review & editing. **Nadine Chan:** Conceptualization, Funding acquisition, Methodology, Writing – review & editing. **Jessica C. Jones-Smith:** Conceptualization, Data curation, Funding acquisition, Methodology, Writing – review & editing.

## Declaration of Competing Interest

The authors declare that they have no known competing financial interests or personal relationships that could have appeared to influence the work reported in this paper.
