# Supplementary material for: Evaluation of Seattle’s sweetened beverage tax on tax support and perceived economic and health impacts
Source: Prev Med Rep. 2022 Apr 30;27:101809. doi: 10.1016/j.pmedr.2022.101809 (PMC9152812; doi:10.1016/j.pmedr.2022.101809)
Supplement: Supplementary data 1 [file mmc1.docx]

**File S1. Survey Questions Related to Tax Support and Tax Impacts in Seattle**

On January 1, 2018, the City of Seattle will/did start taxing sugary drinks. In Seattle, large distributors will/do now pay a 1.75 cents per ounce tax on sugary drinks. Taxed beverages include drinks that have added sugar. The tax will/does NOT include diet beverages, 100% fruit juices, or milk products. Money from the tax will/does help give more people access to healthy and affordable food, expand early education for pre-school aged kids, and help high school graduates enter college.

- 1. Based on what you know, how much do you approve or disapprove of this tax?

🞎 Strongly disapprove

🞎 Somewhat disapprove

🞎 Somewhat approve

🞎 Strongly approve

🞎 Don’t know

- 1. Below is a pair of statements that people have made about this new tax on sugary drinks. Please indicate which statement is closer to your own view, even if neither is exactly right.

| ____ A | **Statement 1:** This tax WILL improve public health in Seattle.  **Statement 2:** This tax will NOT improve public health in Seattle.  🞎 FIRST statement is MUCH closer  🞎 FIRST statement is SOMEWHAT closer  🞎 SECOND statement is MUCH closer  🞎 SECOND statement is SOMEWHAT closer  🞎 Don’t know |
| --- | --- |
| ____ B | **Statement 1:** This tax WILL improve the health and well-being of children in Seattle.  **Statement 2:** This tax will NOT improve the health and well-being of children in Seattle.  🞎 FIRST statement is MUCH closer  🞎 FIRST statement is SOMEWHAT closer  🞎 SECOND statement is MUCH closer  🞎 SECOND statement is SOMEWHAT closer  🞎 Don’t know |
|  |  |

- 1. Below is a pair of statements that people have made about how the new tax on sugary drinks might affect people and businesses in Seattle. Please indicate which statement is closer to your own view, even if neither is exactly right.

| ____ A | **Statement 1:** This tax will have a POSITIVE effect on Seattle's economy.  **Statement 2:** This tax will have a NEGATIVE effect on Seattle's economy.  🞎 FIRST statement is MUCH closer  🞎 FIRST statement is SOMEWHAT closer  🞎 SECOND statement is MUCH closer  🞎 SECOND statement is SOMEWHAT closer  🞎 Don’t know |
| --- | --- |
| ____ B | **Statement 1:** This tax WILL have a negative effect on small businesses in Seattle. Small businesses may lose money and could even go out of business because of the tax.  **Statement 2:** This tax will NOT have negative effects on small businesses in Seattle. It’s not likely that businesses will lose money or go out of business because of the tax.  🞎 FIRST statement is MUCH closer  🞎 FIRST statement is SOMEWHAT closer  🞎 SECOND statement is MUCH closer  🞎 SECOND statement is SOMEWHAT closer  🞎 Don’t know |
| ____ C | **Statement 1:**  This tax WILL result in job loss in Seattle.  **Statement 2:**  This tax will NOT result in job loss in Seattle.  🞎 FIRST statement is MUCH closer  🞎 FIRST statement is SOMEWHAT closer  🞎 SECOND statement is MUCH closer  🞎 SECOND statement is SOMEWHAT closer  🞎 Don’t know |
| ____D | **Statement 1:** This tax WILL have a negative impact on my family's finances  **Statement 2:** This tax will NOT have a negative impact on my family's finances.  🞎 FIRST statement is MUCH closer  🞎 FIRST statement is SOMEWHAT closer  🞎 SECOND statement is MUCH closer  🞎 SECOND statement is SOMEWHAT closer  🞎 Don’t know |
| ____ E | **Statement 1:** This tax will have a POSITIVE impact on people with low-income and people of color’s health and well-being and help them access affordable, healthy food in Seattle.  **Statement 2:** This tax will have a NEGATIVE impact on people with low-income and people of color’s finances, will drive up the cost of living for those who can least afford to pay the tax, and further increase income inequality.  🞎 FIRST statement is MUCH closer  🞎 FIRST statement is SOMEWHAT closer  🞎 SECOND statement is MUCH closer  🞎 SECOND statement is SOMEWHAT closer  🞎 Don’t know |

**File S2. Survey Questions Related to Tax Support and Tax Impacts in the Comparison Area**

Seven U.S. cities will/have now implemented sugary drink taxes. For example, in one city, large distributors will/does now pay a 1.75 cents per ounce tax on sugary drinks. Taxed drinks include drinks that have added sugar. The tax does NOT include diet drinks, 100% fruit juices, or milk products. Money from the tax will help give more people access to healthy and affordable food, expand early education for pre-school aged kids, and help high school graduates enter college.

1. Based on what you know, would you strongly disapprove, somewhat disapprove, somewhat approve, strongly approve of this tax in [city of respondent] ?

🞎 Strongly disapprove

🞎 Somewhat disapprove

🞎 Somewhat approve

🞎 Strongly approve

🞎 Don’t know

1. Below is a pair of statements that people have made about these taxes on sugary drinks. Please indicate which statement is closer to your own view, even if neither is exactly right.

| ____ A | **Statement 1:** These taxes WOULD improve public health.  **Statement 2:** These taxes would NOT improve public health.  🞎 FIRST statement is MUCH closer  🞎 FIRST statement is SOMEWHAT closer  🞎 SECOND statement is MUCH closer  🞎 SECOND statement is SOMEWHAT closer  🞎 Don’t know |
| --- | --- |
| ____ B | **Statement 1:** These taxes WOULD improve the health and well-being of children.  **Statement 2:** These taxes would NOT improve the health and well-being of children.  🞎 FIRST statement is MUCH closer  🞎 FIRST statement is SOMEWHAT closer  🞎 SECOND statement is MUCH closer  🞎 SECOND statement is SOMEWHAT closer  🞎 Don’t know |
|  |  |

| 1. Below is a pair of statements that people have made about these taxes on sugary drinks might affect people and businesses. Please indicate which statement is closer to your own view, even if neither is exactly right. | |
| --- | --- |
| ____ A | **Statement 1:** These taxes WOULD have a POSITIVE effect on the economy.  **Statement 2:** These taxes WOULD have a NEGATIVE effect on the economy.  🞎 FIRST statement is MUCH closer  🞎 FIRST statement is SOMEWHAT closer  🞎 SECOND statement is MUCH closer  🞎 SECOND statement is SOMEWHAT closer  🞎 Don’t know |
| ____ B | **Statement 1:** These taxes WOULD have a negative effect on small businesses. Small businesses may lose money and could even go out of business because of the tax.  **Statement 2:** These taxes would NOT have negative effects on small businesses. It’s not likely that businesses will lose money or go out of business because of the tax.  🞎 FIRST statement is MUCH closer  🞎 FIRST statement is SOMEWHAT closer  🞎 SECOND statement is MUCH closer  🞎 SECOND statement is SOMEWHAT closer  🞎 Don’t know |
| ____ C | **Statement 1:**  These taxes WOULD result in job loss.  **Statement 2:**  These taxes would NOT result in job loss.  🞎 FIRST statement is MUCH closer  🞎 FIRST statement is SOMEWHAT closer  🞎 SECOND statement is MUCH closer  🞎 SECOND statement is SOMEWHAT closer  🞎 Don’t know |
| ____ D | **Statement 1:**  These taxes WOULD have a negative impact on my family's finances  **Statement 2:**  These taxes would NOT have a negative impact on my family's finances.  🞎 FIRST statement is MUCH closer  🞎 FIRST statement is SOMEWHAT closer  🞎 SECOND statement is MUCH closer  🞎 SECOND statement is SOMEWHAT closer  🞎 Don’t know |
| ____ E | **Statement 1:**  These taxes would have a POSITIVE impact on people with low-income and people of color’s health and well-being and help them access affordable, healthy food.  **Statement 2:**  These taxes would have a NEGATIVE impact on people with low-income and people of color’s finances, would drive up the cost of living for those who can least afford to pay the tax, and further increase income inequality.  🞎 FIRST statement is MUCH closer  🞎 FIRST statement is SOMEWHAT closer  🞎 SECOND statement is MUCH closer  🞎 SECOND statement is SOMEWHAT closer  🞎 Don’t know |

**Table S1a: Covariate Balance Among Lower-Income Individuals, Before versus After Weighting^a^**

|  | **Baseline Seattle v. Baseline Comparison** | | | | **Baseline Seattle v. Endline Comparison** | | | | **Baseline Seattle v. Endline Seattle** | | | |
| --- | --- | --- | --- | --- | --- | --- | --- | --- | --- | --- | --- | --- |
|  | No Weight | | PWxPSW | | No Weight | | PWxPSW | | No Weight | | PWxPSW | |
| **Covariate** | SD | VR | SD | VR | SD | VR | SD | VR | SD | VR | SD | VR |
| White | 0.03 | 0.99 | -0.02 | 1.00 | 0.01 | 1.00 | 0.08 | 1.00 | 0.15 | 0.92 | 0.04 | 1.00 |
| Black | 0.00 | 0.99 | 0.01 | 1.02 | 0.09 | **1.23** | -0.05 | 0.89 | -0.07 | 0.84 | -0.03 | 0.94 |
| Asian | -0.16 | **0.60** | 0.01 | 1.02 | -0.05 | 0.86 | -0.08 | 0.87 | 0.08 | **1.34** | -0.07 | 0.88 |
| Other | -0.26 | **0.47** | -0.03 | 0.92 | -0.12 | **0.75** | 0.03 | 1.07 | 0.10 | **1.39** | 0.03 | 1.08 |
| Some HS | 0.09 | **1.37** | -0.11 | **0.76** | -0.17 | **0.43** | -0.08 | 0.83 | -0.22 | **0.40** | 0.06 | 1.18 |
| HS | 0.06 | 1.12 | -0.03 | 0.94 | 0.17 | **1.32** | 0.05 | 1.09 | 0.04 | 1.07 | 0.01 | 1.01 |
| Some College | -0.04 | 0.98 | 0.11 | 1.06 | 0.08 | 1.04 | 0.02 | 1.02 | 0.13 | 1.07 | -0.15 | 0.92 |
| College | -0.07 | 0.93 | -0.02 | 0.98 | -0.04 | 0.96 | -0.07 | 0.92 | 0.01 | 1.01 | 0.08 | 1.08 |
| Graduate | 0.01 | 1.02 | 0.00 | 1.01 | -0.16 | **0.67** | 0.08 | **1.22** | -0.10 | **0.80** | 0.04 | 1.11 |
| <$30,000 | -0.38 | 1.10 | -0.01 | 1.00 | -0.09 | 1.05 | 0.21 | 1.00 | 0.24 | 0.97 | 0.10 | 1.01 |
| $30,000-$59,999 | 0.20 | 1.14 | -0.09 | 0.98 | 0.14 | 1.10 | -0.09 | 0.98 | -0.07 | 0.97 | 0.01 | 1.00 |
| $60,000-$89,999 | 0.35 | **3.41** | 0.18 | **1.75** | -0.08 | **0.64** | -0.21 | **0.35** | -0.27 | **0.43** | -0.12 | **0.70** |
| $90,000-$120,000 | 0.01 | 1.13 | 0.01 | 1.08 | -0.13 | **0.00** | -0.18 | **0.00** | -0.14 | **0.00** | -0.19 | **0.00** |
| 18-30 y | 0.13 | 1.20 | -0.02 | 0.98 | 0.14 | **1.21** | 0.01 | 1.01 | -0.13 | 0.84 | -0.03 | 0.96 |
| 31-40 y | 0.12 | 1.19 | -0.06 | 0.92 | 0.03 | 1.04 | 0.01 | 1.01 | -0.25 | **0.67** | 0.06 | 1.08 |
| 41-50 y | -0.01 | 0.98 | 0.11 | **1.22** | 0.00 | 0.99 | -0.05 | 0.91 | 0.05 | 1.12 | -0.10 | 0.85 |
| 51-64 y | -0.03 | 0.95 | -0.10 | 0.87 | -0.02 | 0.98 | 0.01 | 1.01 | 0.22 | **1.32** | 0.11 | 1.16 |
| 65+ | -0.21 | **0.77** | 0.09 | **1.21** | -0.14 | 0.85 | 0.02 | 1.05 | 0.09 | 1.14 | -0.04 | 0.92 |
| Democrat | -0.29 | 1.01 | 0.01 | 1.00 | -0.17 | 1.03 | 0.02 | 1.00 | 0.13 | 1.02 | 0.00 | 1.00 |
| Independent | 0.16 | 1.17 | -0.03 | 0.97 | -0.01 | 0.99 | 0.02 | 1.02 | 0.01 | 1.00 | 0.06 | 1.08 |
| Republican | 0.15 | **1.54** | -0.05 | 0.84 | 0.07 | **1.23** | -0.02 | 0.95 | -0.13 | **0.70** | 0.06 | **1.23** |
| Other | 0.02 | 1.13 | 0.02 | 1.15 | 0.05 | **1.44** | -0.01 | 0.97 | 0.11 | **1.94** | -0.02 | 0.89 |
| Married | 0.40 | **1.56** | -0.02 | 0.97 | 0.14 | **1.23** | -0.02 | 0.97 | -0.32 | **0.72** | 0.05 | 1.07 |
| Divorced | -0.17 | **0.77** | -0.01 | 0.99 | -0.02 | 0.97 | 0.09 | 1.16 | 0.21 | **1.35** | 0.06 | 1.10 |
| Single | -0.18 | 0.96 | 0.04 | 1.00 | -0.06 | 0.99 | -0.02 | 1.00 | 0.12 | 1.04 | -0.06 | 1.00 |
| Living Together | -0.04 | 0.88 | -0.03 | 0.94 | -0.06 | 0.85 | -0.06 | 0.86 | 0.00 | 0.99 | -0.05 | 0.86 |
| Gender | -0.17 | 1.06 | 0.13 | 0.96 | 0.19 | 0.87 | -0.01 | 1.00 | 0.17 | 0.94 | -0.18 | 1.05 |
| Survey Mode | -0.19 | 0.90 | -0.01 | 0.99 | -0.50 | **0.63** | 0.03 | 1.02 | 0.10 | 1.07 | 0.09 | 1.06 |
| **Average** | **-0.02** | **1.08** | **0.00** | **0.98** | **-0.03** | **0.91** | **-0.01** | **0.90** | **0.01** | **0.97** | **-0.01** | **0.97** |

HS = high school; SD = standardized difference; VR = variance ratio; PW = population weight; PSW = propensity score weight

**^a^** Red bold font indicates VR that are < 0.80 or >1.20. Optimal values: average VR = 1.0 average standardized differences = 0

**Table S1b: Covariate Balance Among Higher-Income Individuals, Before versus After Weighting^a^**

|  | **Baseline Seattle v. Baseline Comparison** | | | | **Baseline Seattle v. Endline Comparison** | | | | **Baseline Seattle v. Endline Seattle** | | | |
| --- | --- | --- | --- | --- | --- | --- | --- | --- | --- | --- | --- | --- |
|  | No Weight | | PWxPSW | | No Weight | | PWxPSW | | No Weight | | PWxPSW | |
| **Covariate** | SD | VR | SD | VR | SD | VR | SD | VR | SD | VR | SD | VR |
| White | -0.33 | **1.43** | 0.05 | 0.94 | -0.31 | **1.42** | -0.04 | 1.05 | 0.08 | 0.94 | -0.04 | 1.05 |
| Black | 0.17 | **2.14** | 0.00 | 1.02 | 0.35 | **3.78** | -0.03 | 0.83 | -0.03 | 0.88 | 0.04 | **1.21** |
| Asian | 0.24 | **2.00** | -0.09 | 0.80 | 0.14 | **1.54** | 0.05 | 1.13 | -0.08 | 0.83 | 0.06 | 1.17 |
| Other | -0.14 | **0.50** | -0.06 | **0.75** | 0.00 | 1.02 | 0.06 | **1.32** | 0.20 | **2.49** | 0.07 | **1.41** |
| Some HS | 0.18 | **9.04** | 0.01 | 1.15 | 0.00 | 1.02 | -0.07 | **0.30** | -0.04 | **0.77** | 0.00 | 0.96 |
| HS | 0.17 | **1.91** | 0.02 | 1.08 | 0.12 | **1.63** | -0.03 | 0.89 | -0.08 | **0.76** | -0.08 | **0.73** |
| Some College | 0.16 | **1.33** | 0.06 | 1.14 | 0.11 | **1.22** | 0.06 | 1.14 | -0.02 | 0.96 | -0.08 | 0.85 |
| College | -0.22 | 0.89 | -0.03 | 0.99 | 0.07 | 1.02 | 0.04 | 1.01 | 0.19 | 1.12 | 0.01 | 1.00 |
| Graduate | -0.02 | 0.99 | -0.02 | 0.99 | -0.22 | 0.87 | -0.07 | 0.96 | -0.12 | 0.93 | 0.08 | 1.05 |
| <$30,000 | 0.16 | **3.00** | 0.20 | **5.12** | 0.00 | 1.02 | -0.03 | **0.71** | -0.14 | **0.40** | -0.17 | **0.29** |
| $30,000-$59,999 | 0.01 | 1.02 | 0.07 | 1.09 | 0.30 | **1.30** | 0.14 | 1.17 | 0.11 | 1.12 | -0.03 | 0.97 |
| $60,000-$89,999 | -0.10 | 0.89 | -0.10 | 0.90 | 0.16 | 1.14 | 0.13 | 1.11 | 0.18 | **1.21** | 0.08 | 1.09 |
| $90,000-$120,000 | 0.03 | 1.04 | 0.07 | 1.10 | -0.13 | 0.80 | 0.00 | 1.00 | -0.05 | 0.92 | 0.00 | 1.00 |
| > $120,000 | 0.00 | 1.00 | -0.10 | 0.91 | -0.41 | **0.51** | -0.27 | **0.72** | -0.20 | **0.78** | 0.01 | 1.01 |
| 18-30 y | 0.06 | 1.13 | 0.00 | 1.00 | 0.27 | **1.54** | 0.07 | 1.12 | 0.03 | 1.05 | 0.00 | 1.00 |
| 31-40 y | 0.10 | 1.16 | 0.09 | 1.12 | 0.10 | 1.16 | 0.00 | 1.00 | 0.06 | 1.08 | -0.10 | 0.89 |
| 41-50 y | -0.09 | 0.85 | -0.14 | 0.81 | -0.18 | **0.70** | -0.12 | 0.84 | 0.07 | 1.14 | 0.08 | 1.14 |
| 51-64 y | 0.01 | 1.01 | 0.08 | 1.09 | -0.06 | 0.91 | -0.01 | 0.99 | 0.06 | 1.09 | -0.06 | 0.93 |
| 65+ | -0.07 | 0.94 | -0.04 | 0.92 | -0.13 | 0.87 | 0.06 | 1.12 | -0.21 | **0.76** | 0.10 | **1.21** |
| Democrat | -0.23 | 0.99 | 0.03 | 1.00 | -0.05 | 1.01 | -0.02 | 1.00 | 0.29 | 0.99 | 0.04 | 0.99 |
| Independent | -0.05 | 0.96 | 0.00 | 1.00 | -0.17 | 0.85 | -0.03 | 0.98 | -0.11 | 0.90 | -0.06 | 0.95 |
| Republican | 0.32 | **2.00** | -0.04 | 0.89 | 0.19 | **1.59** | 0.05 | 1.15 | -0.25 | **0.61** | 0.05 | 1.17 |
| Other | 0.06 | **1.63** | -0.01 | 0.95 | 0.00 | 1.02 | 0.06 | **1.62** | 0.07 | **1.59** | 0.00 | 1.00 |
| Married | -0.02 | 1.00 | -0.10 | 1.01 | -0.48 | 0.87 | -0.07 | 1.01 | -0.27 | 0.97 | 0.10 | 0.99 |
| Divorced | -0.05 | 0.90 | 0.00 | 1.01 | 0.10 | **1.21** | 0.00 | 1.00 | -0.01 | 0.97 | 0.02 | 1.07 |
| Single | 0.09 | 1.11 | 0.06 | 1.06 | 0.45 | **1.39** | 0.06 | 1.06 | 0.21 | 1.18 | -0.07 | 0.94 |
| Living Together | -0.06 | 0.84 | 0.07 | **1.22** | -0.03 | 0.91 | 0.02 | 1.06 | 0.14 | **1.50** | -0.09 | **0.77** |
| Gender | -0.35 | 0.96 | 0.01 | 1.00 | 0.18 | 0.93 | 0.01 | 1.00 | 0.31 | 1.05 | 0.07 | 1.01 |
| Survey Mode | -0.75 | **0.57** | -0.04 | 1.00 | -0.75 | **0.57** | -0.03 | 1.00 | 0.26 | **1.42** | 0.05 | 1.01 |
| **Average** | **-0.02** | **1.49** | **0.00** | **1.14** | **-0.01** | **1.17** | **0.00** | **1.01** | **0.02** | **1.05** | **0.00** | **0.99** |

HS = high school; SD = standardized difference; VR = variance ratio; PW = population weight; PSW = propensity score weight

**^a^** Red bold font indicates VR that are < 0.80 or >1.20. Optimal values: average VR = 1.0 average standardized differences = 0.

| **Table S2. Descriptive Pre-tax to Post-tax Prevalences in Perceptions of Sweetened Beverage Taxes in Seattle, Washington, and the Comparison areas, by Income** | | | | | | | | | | | | | | | | |
| --- | --- | --- | --- | --- | --- | --- | --- | --- | --- | --- | --- | --- | --- | --- | --- | --- |
|  | Lower-Income^a^ | | | | | | | | Higher-Income^b^ | | | | | | | |
|  | Seattle | | | | Comparison | | | | Seattle | | | | Comparison | | | |
|  | Pre-tax^b^ | | Post-tax^b^ | | Pre-tax^b^ | | Post-tax^b^ | | Pre-tax^b^ | | Post-tax^b^ | | Pre-tax^b^ | | Post-tax^b^ | |
|  | N | % | N | % | N | % | N | % | N | % | N | % | N | % | N | % |
| Support for sugary beverage tax(es) | | | | | | | | |  |  |  |  |  |  |  |  |
| Yes | 202 | 50.4% | 159 | 47.9% | 186 | 60.1% | 148 | 46.5% | 286 | 62.2% | 260 | 61.4% | 232 | 57.7% | 248 | 58.8% |
| No | 161 | 45.5% | 175 | 48.0% | 148 | 36.4% | 168 | 45.8% | 149 | 33.9% | 167 | 36.8% | 186 | 38.4% | 165 | 37.2% |
| Don’t Know | 16 | 4.1% | 16 | 4.2% | 30 | 3.4% | 37 | 7.7% | 19 | 3.9% | 12 | 1.7% | 23 | 3.9% | 25 | 4.0% |
| Tax(es) will/would have negative effects on small businesses | | | | | | | | | | | | | | | | |
| Yes | 153 | 39.9% | 168 | 44.7% | 154 | 34.5% | 171 | 51.7% | 170 | 37.8% | 212 | 50.3% | 192 | 42.4% | 183 | 41.1% |
| No | 187 | 50.6% | 135 | 45.1% | 166 | 57.0% | 124 | 38.4% | 246 | 54.2% | 174 | 40.5% | 202 | 48.7% | 197 | 49.7% |
| Don’t Know | 40 | 9.5% | 47 | 10.3% | 46 | 8.6% | 58 | 9.9% | 37 | 8.0% | 53 | 9.3% | 47 | 8.9% | 58 | 9.1% |
| Tax(es) will/would have a positive effect on the economy | | | | | | | | | | | | | | | | |
| Yes | 156 | 43.0% | 156 | 44.4% | 164 | 48.4% | 150 | 46.1% | 211 | 49.9% | 223 | 50.6% | 198 | 49.5% | 203 | 41.5% |
| No | 143 | 37.6% | 118 | 37.7% | 133 | 37.1% | 122 | 37.2% | 158 | 34.5% | 124 | 31.7% | 162 | 36.8% | 133 | 38.7% |
| Don’t Know | 77 | 19.4% | 76 | 18.0% | 68 | 14.5% | 81 | 16.7% | 85 | 15.6% | 92 | 17.7% | 80 | 13.7% | 102 | 19.8% |
| Tax(es) will/would result in job loss | | | | | | | | | | | | | | | | |
| Yes | 92 | 23.4% | 93 | 24.0% | 114 | 25.8% | 106 | 26.2% | 94 | 21.7% | 114 | 27.2% | 133 | 26.3% | 101 | 20.1% |
| No | 233 | 62.2% | 187 | 60.9% | 196 | 65.6% | 174 | 58.1% | 321 | 69.8% | 254 | 59.4% | 242 | 62.6% | 257 | 67.1% |
| Don’t Know | 55 | 14.4% | 70 | 15.1% | 56 | 8.6% | 73 | 15.7% | 38 | 8.4% | 71 | 13.3% | 66 | 11.1% | 80 | 12.8% |
| Tax(es) will/would have a negative impact on family's finances | | | | | | | | | | | | | | | | |
| Yes | 90 | 25.6% | 119 | 32.0% | 119 | 27.5% | 118 | 31.4% | 55 | 13.5% | 109 | 23.7% | 99 | 19.9% | 76 | 17.9% |
| No | 269 | 66.5% | 211 | 64.4% | 226 | 69.7% | 202 | 64.8% | 392 | 85.1% | 312 | 72.9% | 319 | 76.6% | 329 | 77.0% |
| Don’t Know | 21 | 7.8% | 20 | 3.6% | 21 | 2.8% | 33 | 3.8% | 7 | 1.4% | 18 | 3.4% | 23 | 3.5% | 33 | 5.1% |
| Tax(es) will/would have a positive impact on people with low-income and people of color’s health/well-being | | | | | | | | | | | | | | | | |
| Yes | 168 | 43.9% | 132 | 41.1% | 165 | 50.7% | 138 | 42.6% | 221 | 50.9% | 190 | 44.2% | 177 | 39.5% | 198 | 42.0% |
| No | 164 | 42.5% | 167 | 45.7% | 147 | 33.2% | 158 | 46.2% | 181 | 40.0% | 190 | 44.3% | 215 | 50.9% | 174 | 45.1% |
| Don’t Know | 46 | 13.6% | 51 | 13.2% | 54 | 16.1% | 57 | 11.1% | 50 | 9.1% | 59 | 11.5% | 49 | 9.6% | 66 | 13.0% |
| Tax(es) will/would improve public health | | | | | | | | | | | | | | | | |
| Yes | 186 | 48.8% | 163 | 47.5% | 201 | 63.4% | 168 | 49.4% | 267 | 61.1% | 244 | 60.1% | 240 | 58.2% | 252 | 60.3% |
| No | 174 | 44.8% | 156 | 47.1% | 144 | 33.5% | 151 | 45.2% | 172 | 36.1% | 177 | 37.0% | 177 | 38.8% | 168 | 36.6% |
| Don’t Know | 20 | 6.4% | 31 | 5.4% | 21 | 3.1% | 34 | 5.4% | 15 | 2.7% | 18 | 2.8% | 23 | 3.0% | 18 | 3.1% |
| Tax(es) will/would improve child wellbeing | | | | | | | | | | | | | | | | |
| Yes | 210 | 54.4% | 174 | 49.3% | 209 | 61.0% | 196 | 57.0% | 275 | 62.2% | 264 | 63.7% | 257 | 60.4% | 267 | 64.1% |
| No | 153 | 40.3% | 138 | 42.3% | 131 | 35.6% | 126 | 39.2% | 164 | 35.1% | 160 | 33.8% | 166 | 36.2% | 154 | 33.0% |
| Don’t Know | 17 | 5.3% | 38 | 8.3% | 25 | 3.5% | 31 | 3.7% | 15 | 2.7% | 15 | 2.6% | 17 | 3.3% | 17 | 2.8% |

^a^Lower income is defined as < 260% FPL. Higher income is defined as $\geq$ 260% FPL

^b^ N is unweighted to show the sample size whereas percentages (%) are weighted using the population weight X propensity score weight.
